# A negative role for master regulator Bcr1 in *Candida albicans* hypha-associated gene expression

**DOI:** 10.64898/2026.09.03.749130

**Published:** 2026-09-07

**Authors:** Anupam Sharma, Aaron P. Mitchell

## Abstract

Filamentation, a central virulence trait of the fungal pathogen *Candida albicans,* is required for host cell damage, tissue invasion, and biofilm formation. The transcription factor Bcr1 was first found to be required for biofilm formation but not filamentation. Subsequent studies revealed a negative role for Bcr1 in filamentation in opaque cells, a cell type that is required for mating, under hypoxic conditions, and in a *wor2* Δ/Δ mutant background. Here we characterize a new context in which Bcr1 negatively regulates filamentation and present its associated gene expression impact. We compared the wild-type and *bcr1* Δ/Δ mutant cells in the SC5314 reference strain background at 30°C in glucose media, conditions that do not induce filamentation. The *bcr1* Δ/Δ mutant displayed increased invasive growth in a solid medium, and some filamentation ability in a liquid medium. The *bcr1* Δ/Δ effect on filamentation was augmented by overexpression of hyphal cyclin gene *HGC1,* which caused pseudohyphal growth in wild-type, *efg1* Δ/Δ, or *brg1* Δ/Δ strains, yet caused hyphal growth in the *bcr1* Δ/Δ strain. RNA-sequencing (RNA-seq) analysis of 30°C cells shows that the *bcr1* Δ/Δ mutant has elevated expression of two genes that drive filamentation: *HGC1* and *UME6.* In published data these same two genes have decreased expression in the *bcr1* Δ/Δ mutant at 37°C, the temperature at which biofilm formation is typically assayed. A growing cadre of biofilm/hyphal regulators can have both positive and negative effects on hypha-associated gene expression, including Bcr1, Efg1, Ndt80, and Nrg1.

**IMPORTANCE:**

Production of filamentous cells is a central virulence trait of the fungal pathogen *Candida albicans* . Evidence here shows that the transcription factor Bcr1 is a negative regulator of filamentation at low temperature (30°C). Bcr1 affects expression of two drivers of filamentation, the genes *HGC1* and *UME6.* Surprisingly, Bcr1 inhibits their expression at 30°C but stimulates their expression at 37°C. A growing number of filamentation regulators seem to function as both positive and negative regulators of filamentation, depending upon genetic and environmental contexts.

## Full Text Availability

The license terms selected by the author(s) for this preprint version do not permit archiving in PMC. The full text is available from the preprint server.

